# Cleft of the Hard and Soft Palate Closed by Operation, with Production of Bone in the Palatine Vault

**Published:** 1880-12

**Authors:** A. E. Smith

**Affiliations:** Darlington, Wis.


### Clinical Reports.

### NOTES FROM PRIVATE PRACTICE.

### ARTICLE VI.

*Cleft of the Hard and Soft Palate Closed by Operation with Production of Bone in the Palatine Vault.*

C. L. presented himself at my office, Feb. 8, 1876, aged twenty-two, robust and of good constitution, to be treated for an increasing deafness which had troubled him for three years.

On examination, I found a cleft of the hard and soft palate with considerable angina which I attributed to the fissure, and advised an operation to close it in the hope of improving his hearing as well as speech. The latter was so imperfect and nasal that even his own relatives could understand but little of what he said. The cleft was complete, extending forward to the incisor teeth, and was of the unilateral form. It was also complicated by hare-lip, which was operated upon when he was two months old with good success, a statement being then made to the effect that nothing could be done for the fissure. The cleft was very wide behind, and tapered to almost a point in front. The operation I performed March 15, 1876, assisted by Dr. W. M. Blair. The patient was seated in an adjustable chair in front of a good light, and *no* anæsthetic was used.

Whitehead's gag was employed to keep the mouth open and the tongue out of the way. The palato-pharyngeal muscles were seized low down and divided with a pair of long curved scissors. Then the levator palati muscles of each side were thoroughly divided, also the muco-periosteal membrane lining the lower part of the internal pterygoid plates with Whitehead's knives. After this the side incisions were made through the muco-periosteal tissue of the gums to the bone. These incisions extended from a little back of the last molar to the incisors on each side. The next step was to divide the muco-periosteal tissue along the edge of the cleft of the hard palate to the bone, as well as the palatal muscles along the posterior border of the palate bone, which was done with Whitehead's hoe. After this, I detached the periosteal tissue from the palatal process of the superior maxillary on each side, avoiding the anterior palatine foramen and posteriorly, where the superior palatine artery emerges, with a periosteotome. The paring of the edges of the cleft was done with a long straight-edged knife, holding the flaps with the long curved forceps used for seizing the palato-pharyngeal muscles.

Six silver wire sutures were then passed, three farthest back with Whitehead's medium-sized staphyloraphy needle, three in front with small needles in ordinary needle forceps. After all were passed, I commenced in front and carefully adjusted them. Then the lateral cuts were thoroughly stuffed with lint, which was removed and freshly applied every day after thorough cleansing of both wound and mouth with a weak solution of carbolic acid. He was kept on soups and liquid food for ten days, at which time the union was found complete in its entire extent, except a small aperture about the size of a silver probe at the anterior part of the cleft. The sutures were all removed on the eleventh day, as was also the lint in the side cuts. The opening that remained kindly healed after making three applications of solid nitrate of silver to it, about one week apart. The new palate was sufficiently long and lax to perform well its functions. Returning to my office within two months from the time of operation, the speech was found to be very much improved; in fact, the patient could talk quite plainly. There was also very decided improvement in hearing, so that he could conduct ordinary conversation very well. He was last seen Aug. 1, 1876. Then the situation of the cleft was entirely healed, and, testing with needles, I found the site of the cleft of the hard palate gave the sensation of bone, and the soft palate and uvula were almost of normal appearance.

The especial points in the case were the thorough relaxation of the soft palate and the dissection of the flaps, so that there was sufficient relaxation. With these precautions there will be no tension after union, upon which depends the improvement of speech.

A. E. SMITH, M.D.

DARLINGTON, WIS., Nov. 10, 1880.

---

OXALATE OF CERIUM IN THE TREATMENT OF CHOLERA INFANTUM.—A country practitioner writes to the *Concours Médicale* on the treatment of infantile cholera by oxalate of cerium. He says: "Profiting by a slight epidemic of diarrhœa among children and adults, I administered oxalate of cerium regularly, in doses indicated by my *confrére*, Dr. Poulet, viz., four grains divided into ten powders for children under two years, one to be given every hour; eight grains in ten powders between two and ten years; and fifteen grains similarly divided for adults. Without any exception, I have obtained rapid and absolute cessation of the vomiting, and in four cases, complete cure of the diarrhœa. In a child of a month old, already much attenuated by the disease, I renewed, during three days successively, the dose of four grains in ten powders; each time I obtained the cessation of the vomiting, which returned when the medicine was discontinued. Unfortunately, it was impossible for me to arrest the diarrhœa, and the child succumbed; but I am convinced that if I had been called in earlier I could have saved it. I cannot impress too strongly the importance of the cessation of the vomiting, as it permits the employment of the administration of the remedies usual in these cases. I tried to give the oxalate of cerium in a mixture, but I had to abandon it in favor of the powders. In a word, the employment of oxalate of cerium seems to me indicated in infantile cholera, and the cholera nostras of adults at the vomiting period. Its action upon the stomach is incontestable; and if that which it exercises on the intestine is more doubtful, it permits, at least, the recourse to other medicines the administration of which would have been otherwise impossible."—*Med. and Surg. Reporter.*

